# Perceived influence of oral health upon quality of life in heart transplant patients

**DOI:** 10.4317/medoral.17542

**Published:** 2011-12-06

**Authors:** Rafael Segura-Saint-Gerons, Carmen Segura-Saint-Gerons, Rosario Alcántara-Luque, José M. Arizón-del Prado, Carla Foronda-Garcia-Hidalgo, Antonio Blanco-Hungría

**Affiliations:** 1 DDS. La Carlota Primary Care Center. Guadalquivir District. Córdoba. Andalusian Health Service; 2 Nurse. Heart Transplantation Unit. Reina Sofía University Hospital. Andalusian Health Service; 3 Nurse. Centro Levante Sur Primary Care Center. Córdoba Center District. Andalusian Health Service; 4 Cardiologist. Reina Sofía University Hospital. Andalusian Health Service; 5 Resident in Primary Care. Posadas Primary Care Center. Guadalquivir District. Córdoba. Andalusian Health Service; 6 DDS. Avenida Aeropuerto Primary Care Center. Córdoba District. Córdoba. Andalusian Health Service (Spain)

## Abstract

Objective: A study was made of heart transplant patient perception of the influence of oral health upon quality of life, based on the Oral Health Impact Profile (OHIP-49) questionnaire validated for Spanish speaking subjects. Design: A cross-sectional evaluation was made of the heart transplant patients followed-up on in the Heart Transplantation Unit of Reina Sofía University Hospital (Spain), using the OHIP-49 questionnaire. The included patients were all over age 18 and signed the corresponding informed consent to participation in the study. The data were entered in a database and analyzed using the SPSS statistical package. Results: A total of 150 heart transplant patients (118 males and 32 females, with a mean age of 54.94 years; range 19-79) were studied. The subjects showed a poor perceived influence of oral health upon quality of life, with a mean score of 24.43 out of a possible total of 196 points. Women showed significantly improved perception of the influence of oral health upon quality of life versus men. Conclusions: The subjects in our study showed a poor perceived influence of oral health upon quality of life.

** Key words:**Perception, oral health, quality of life, heart transplant.

## Introduction

In the industrialized world, thanks to progressive improvements in the prevention, diagnosis and treatment of diseases, it has been possible to reduce the morbidity and mortality associated with a range of disorders – prolonging patient life expectancy but at the same time increasing the presence of nonfatal, chronic degenerative processes that currently represent the main public health problem in these countries.

In this context, it does not seem advisable to assess population survival in strict terms of life expectancy. In effect, in the industrialized world this term has become obsolete, since it tells us nothing of the capacity of patients to go about their daily life activities, or of their physical, mental and emotional wellbeing – i.e., in sum, of their quality of life (1).

Oral quality of life indicators were developed in the 1970s to assess the physical, psychological and social impact of oral problems and to complement the existing clinical indicators, since the latter are insensitive to subjective perceptions such as pain, aesthetics or function (2).

In 1993 the World Health Organization (WHO) defined quality of life as “individual perception of the degree of pleasure in life, taking into account personal desires, expectations and paradigms, in accordance with the system of values found in the socio-cultural context of the subject” (3).

Thanks to advances in many areas of Medicine, survival among heart transplant patients has improved spectacularly. Thus, according to the Spanish Heart Transplant Registry, survival after the first year of transplantation is 76%, and 8 years after transplantation the mean life expectancy is 11 years (4).

Although transplantation greatly improves survival, the patients are exposed to a series of conditioning factors (the need for immune suppressive medication for life, and the possibility of rejection, infections or tumors) that make it necessary not only to assess their physical condition through objective tests but also their psychological or emotional state by means of tests designed to assess the quality of life of these individuals.

According to White and Williams, the components of quality of life are classified into four domains: physical and mental, functional capacity, psychological capacity, and social interactions (5).

Tests have been developed to assess these aspects, involving items that measure all the categories or domains (general tests), or which focus on a concrete category (specific tests).

As generic quality of life questionnaires, mention can be made of the Nottingham Health Profile, the SF-36 or Lough quality of life questionnaire. The specific tests in turn include the dyspnea and fatigue scale, or the heart transplantation symptoms list (6).

Many questionnaires have been designed to examine the impact of oral health upon quality of life since Cushing in 1986 developed the Social Impact of Oral Disease test. Thus, the Dental Impact on Daily Living questionnaire was introduced in 1995, while the Dental Impact Profile was developed in 1997 (7).

In 1994, Slade and Spencer designed the Oral Health Impact Profile (OHIP-49), comprising 49 items used to assess the four quality of life dimensions of White and Williams (8). This instrument was posteriorly translated and validated in its Spanish version by López and Baelum in 2006 (9,10).

On occasion of the closing session of the XLIV Congress of the Spanish Society of Periodontics held in Girona, the cardiologist Valentín Fuster made the following observation: “I doubt whether someone who is unable to care for his gums is able to take care of other living habits and follow minimum healthy life recommendations, or vice versa”. This expert therefore insisted on the “need to promote oral health, since to do so will promote the modification of risk factors common to other diseases”.

To date, no studies have examined the impact of oral health in heart transplant patients, though if good quality of life is to be reached in such individuals, the study of this subject must be extended to all patient capacities, with a view to improving those aspects of our healthcare services that show weaknesses.

## Material and Methods

A cross-sectional study was made of heart transplant patient perception of oral health, using the Oral Health Impact Profile (OHIP-49) questionnaire. The validated Spanish version of this instrument explores patient perceived quality of life related to oral health, based on 49 questions divided into 7 different domains. Each question is answered using a Likert-type scale scored from 0-4 points as follows: 0 = never, 1 = almost never, 2 = sometimes, 3 = quite often, 4 = very often.

The questionnaire assesses 7 domains, each with a score range dependent upon the number of items involved: 1.- Functional limitation (9 items): possible range 0-36; 2.- Physical pain (9 items): possible range 0-36; 3.- Psychological discomfort (5 items): possible range 0-20; 4.- Physical incapacitation (9 items): possible range 0-36; 5.- Psychological incapacitation (6 items): possible range 0-24; 6.- Social incapacitation (5 items): possible range 0-20; and 7.- Disability (6 items): possible range 0-24.

The OHIP-49, in contrast to other quality of life questionnaires, yields a final summarizing score (contemplating all the items), reflecting better or worse patient perception of oral quality of life: 49 (items) x 0-4 (possible range of the Likert-type scale) = 0 (poorer perception of the influence of oral health upon quality of life) / 196 (improved perception of the influence of oral health upon quality of life).

The inclusion criteria of the study were: (a) heart transplant patients visiting the center for clinical follow-up; (b) patients over 18 years of age who signed the corresponding informed consent. The study protocol was approved by the Ethics Committee of Reina Sofia University Hospital.

The questionnaire was self-administered, except for illiterate patients, where one same investigator in all cases conducted the interview.

Personal data were recorded in all cases, including patient age, gender, social and educational level, background disease, transplant code (programmed or emergency), and years from transplantation. In addition, we documented behavioral information relating to habits of hygiene and dental revisions, and all aspects that could influence patient perception of oral health.

In estimating the sample size, we considered the expected proportion of patients reporting oral health to influence their quality of life to be 30% (as recorded in a previous study on quality of life) with a 95% confidence interval and an error of 3% - the resulting required sample size being 150 patients.

The data were entered in a database and analyzed with the SPSS version 17.0 statistical package. The variables were subjected to a descriptive study, calculating the absolute and percentage frequencies for qualitative variables, and the arithmetic mean, standard deviation (SD) and range for quantitative variables.

A descriptive analysis was also made of the variable obtained from the results of the questionnaire. This variable was of a quantitative nature (the sum of the scores of the 49 questions of the OHIP-49), and the corresponding arithmetic mean informed us of the patient perceived importance of oral health in relation to quality of life, based on a scale of 0-196.

Bivariate analysis using the opportune parametric and nonparametric contrasts was performed to relate the dependent variable to the rest of variables.

Lastly, multiple linear regression analysis was carried out to relate the principal variable to the rest of the variables and control possible confounding factors and interactions.

All contrasts were two-tailed, statistical significance being accepted for p ≤ 0.05.

A multiple linear regression model was used to identify the factors associated to patient perceived oral health, incorporating the following variables: age (years), gender (1 = male, 2 = female), years from transplantation (1 = more than 5 years, 2 = less than 5 years), number of visits to the dentist, and number of tooth brushings daily.

The variables with p ≥ 0.05 for the Student t-statistic were subjected to stepwise backward elimination from the model. Comparison of the resulting reduced model with the model including the eliminated variables was carried out with the multiple partial F test.

The scales of the continuous variables were assessed by means of the Box Tidwell test, examining the possible interactions. The variables with p > 0.05 were taken to be possible confounding factors if the percentage change of the coefficients exceeded 15%.

Colinearity among independent variables was assessed by the variance inflation factor (VIF). Independence and homoscedasticity of the residuals of the model were analyzed with the Durbin-Watson test and the dispersion plot between the residuals and the estimated values, respectively. As diagnostic test for identifying extreme cases (outliers), use was made of Cook’s distance.

The corrected coefficient of determination (R2) was used to assess the goodness of fit, since it expresses the proportion of variance of the dependent variable explained by the independent variables – values close to 1 indicating improved fit – though the purpose of the model was associate, not predictive.

The problem raised in conducting this study is inherent to the questionnaire employed. In effect, while the instrument has been validated for Spanish-speaking subjects, the translation poses difficulties in understanding some of the questions, and some of these are moreover repetitive.

Interviewer bias, as an example of information bias, can occur when the field personnel know the condition of the interviewed patient, and if they moreover know the study hypothesis or objectives. In order to avoid this problem, the questionnaire was self-administered, except for illiterate patients, where one same investigator in all cases conducted the interview.

## Results

The OHIP-49 was applied to a total of 150 patients, of which 118 were men (78.7%) and 32 women (21.3%). The mean age was 54.94 years (standard deviation 14.56)(range 19-79 years).

The indications for heart transplantation were dilated myocardiopathy in 71 cases (47.3%), ischemic heart disease in 49 (32.7%), vascular disease in 11 (7.3%) and other causes in 8 patients (5.3%). Transplantation was programmed in 125 cases (83.3%), while emergency transplantation was carried out in 25 cases (16.7%)( Table 1).

**Table 1:**
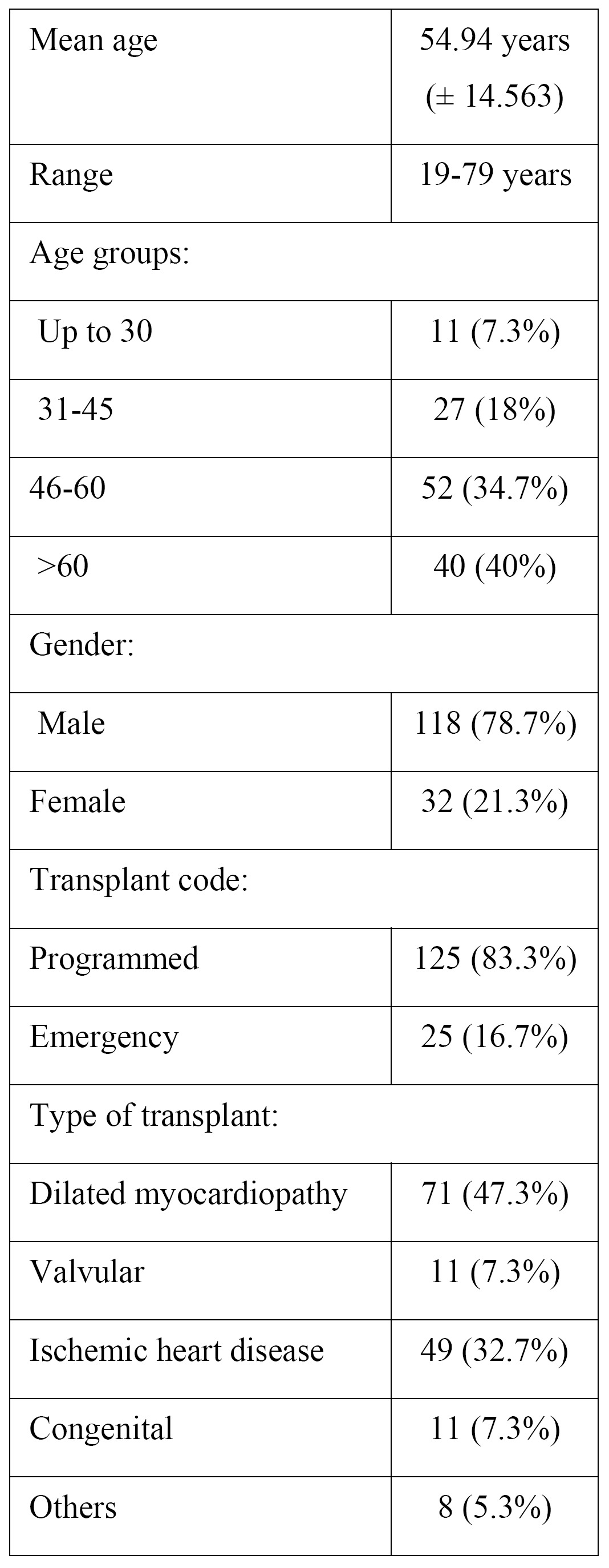
Basal characteristics of the study population.

The time from transplantation was less than 5 years in 46 patients (30.7%), between 5 and 10 years in 48 patients (32 %) and over 10 years in 56 patients (37.3%).

Regarding oral hygiene, 12 patients (8%) did not brush their teeth, 43 (28.7%) brushed once a day, 46 (30.7%) twice a day, 47 (31.3%) three times a day, and two patients (1.3%) brushed their teeth four times a day.

In the last year 80 patients (53.3%) had not visited the dentist, while 29 (19.3%) had visited once, 20 (13.3%) had visited twice, and 21 (14 %) had visited the dentists three or more times.

The reasons for visiting the dentist were tartrectomy in 24 patients (16%), fillings in 14 (9.3%), prostheses in 8 (5.3%), extractions in 12 (8%) and other reasons in 12 patients (8%).

Eighty-nine patients (62%) wore no dentures, while 61 (49.7%) wore dentures: fixed in 13 cases (59.3%), removable (resin) in 32 (21.3%) and removal (skeletal) in 16 (10.7%).

The OHIP-49 yielded a mean score of 24.43 points out of a possible total of 196 points. Regarding the 7 studied domains, physical pain yielded a mean score of 6.82 out of a total of 36 possible points, with a standard deviation of 6.723 and a range of 0-32 points. This was followed by functional limitation with a mean score of 6.5 out of a total of 36 possible points, a standard deviation of 5.710 and a range of 0-29; psychological discomfort with a mean score of 3.42, a standard deviation of 4.281 and a range of 0-19; physical incapacitation with a mean score of 3.31, a standard deviation of 5.038 and a range 0-29; psychological incapacitation with a mean score of 2.31, a standard deviation of 4.055 and a range of 0-24; social incapacitation with a mean score of 0.83, a standard deviation of 2.337 and a range of 0-17; and finally disability with a mean score of 1.24, a standard deviation of 2.514 and a range of 0-19.

The bivariate analysis of the different variables using the Student t-test only identified significance for the variable gender and the mean global score of the interview – females showing a greater mean score than males (p= 0.008). Likewise, women suffered comparatively greater functional limitation (p=0.027) and greater physical pain (p=0.021) and disability (p=0.030).

According to the linear regression model ( Table 2), the variables that finally influenced the questionnaire results were the number of daily brushings, the visits to the dentist in the last year, and patient gender – interaction being observed between gender and the number of visits to the dentist.

**Table 2:**
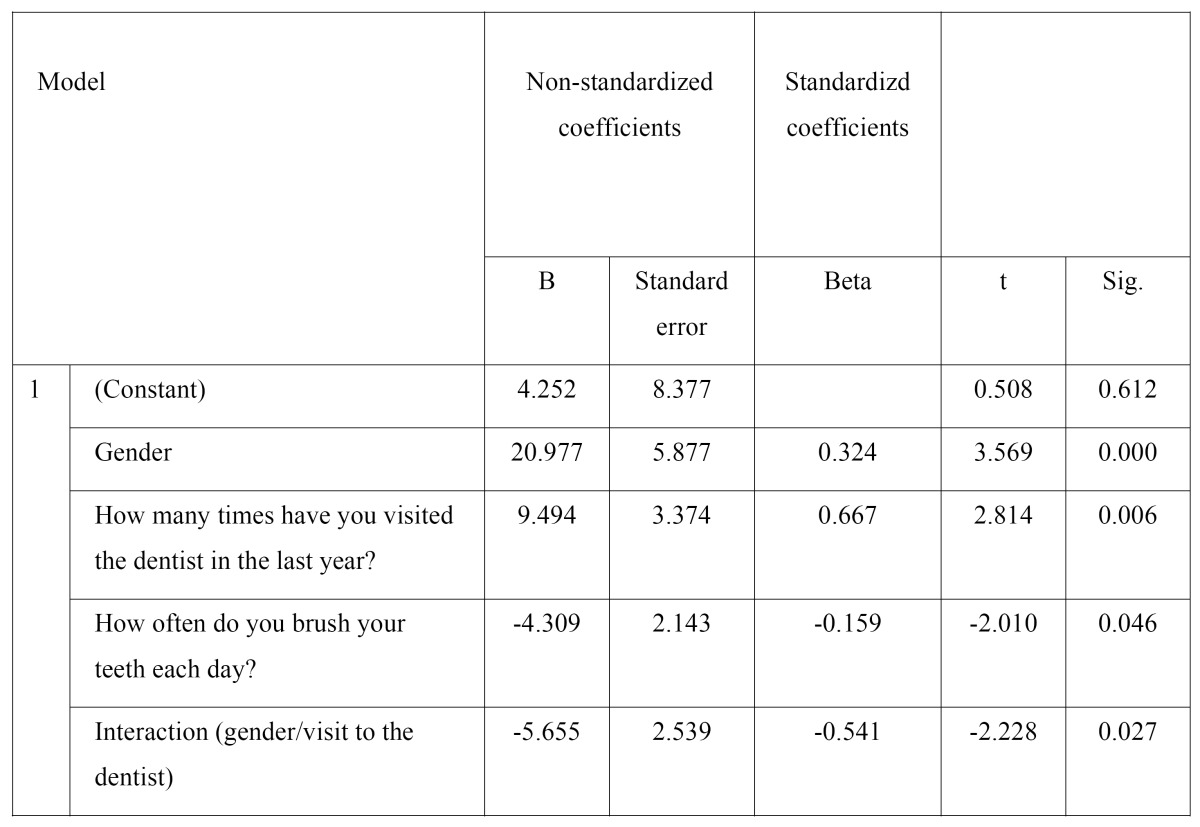
Linear regression model.

Tooth brushing one time less often implied a reduction in the questionnaire score of 4.31 (p=0.046) – this possibly indicating that people who brush their teeth more often place greater importance on oral health in relation to quality of life, for equal gender and visits to the dentist.

In order to assess the influence of gender upon the final questionnaire score, consideration is required of the number of visits to the dentist, since these two variables were found to interact. Results are thus obtained according to low, intermediate or high values corresponding to the variable visits to the dentist.

In people who have never visited the dentist, the female gender implied an increase in the final score of 20.98 points, which indicates that women place more importance on oral health than men.

In people who have visited the dentist twice in the last year (intermediate value), women were seen to continue to place greater importance on oral health than men, with an increase in the final score of 9.67 points.

Lastly, in people who have visited the dentist 5 times in the last year, the male gender was associated to increased concern about oral hygiene, with an increase in the final score of 7.29 points.

Patient gender is therefore seen to exert an influence when interpreting the variable visits to the dentist. In this context, the women who visit the dentist more often showed an increase in the questionnaire score of 7.68 points versus women who do not visit the dentist, for an equal number of daily tooth brushings. In turn, the men who visit the dentist more often placed greater importance on oral health, as evidenced by an increase of 3.84 points in the OHIP-49 questionnaire.

## Discussion

The study population comprises a significant sample of the heart transplant patients in Reina Sofía University Hospital (Spain). The results therefore can be extrapolated to the reference population, though multicenter studies are needed in order to extend the findings to the global heart transplant population.

Oral health has been an important factor in relation to patient quality of life (11). Administration of the OHIP-49 has posed difficulties due to the great extent of the questionnaire. In this context we consider that it would be interesting to carry out the study using the validated short-version OHIP-14 (12), and examining the possible existence of differences in the results obtained.

The global questionnaire score has been low in our study (24.43 points out of 196), indicating that heart transplant patient perception regarding the influence of oral health upon quality of life is low. Although these subjects receive instructions on oral health before and after transplantation, our patients did not seem to be particularly worried about their oral health – possibly because of greater concern regarding their other treatments, or due to the spectacular improvement in quality of life as a result of transplantation.

These observations are supported by the results obtained in the domains relating to oral pain and functional limitation, where the scores were highest – indicating that the patients became concerned about their oral health when complications developed as a result of a lack of oral care.

Important gender differences were recorded, since women showed increased perception of the importance of oral health in relation to quality of life when visiting the dentist between 0-2 times a year, and keeping the rest of the variables constant. However, when the dentist was visited more frequently (5 times a year), men were seen to place comparatively greater importance on oral health in relation to quality of life.

The detection of an interaction between visits to the dentist and patient gender provides a new area for further research, since as has been seen, both variables influence oral health, and therefore also quality of life.

Health-related quality of life is a very promising outcome measure in studies of effectiveness. Its use allows us to go beyond solid and objective measures which are nevertheless limited to very notorious phenomena or events such as mortality or myocardial infarction rate – offering more subtle information referred to less apparent events but which are of great importance for assessing the health conditions of a given population.

No studies to date have correlated oral health and quality of life in transplant patients. This may be due to a lack of awareness among healthcare professionals regarding the possible oral complications of transplant patients, and which lead to a decrease in quality of life.

## Conclusions

The heart transplant patients in our study showed a poor perceived influence of oral health upon quality of life.

In the present century patients will become the most important emerging protagonists, with an increased decision taking capacity in the different healthcare systems throughout the world. In this context it will be crucial to determine how the different diseases and treatments influence their quality of life.

The lack of studies on the subject encourages us to continue working in this field. In this sense we consider it important for other research groups to carry out studies for comparison purposes, with a view to drawing conclusions that can be extrapolated to larger population groups.
